# Novel Effects of Hormonal Contraceptive Use on the Plasma Proteome

**DOI:** 10.1371/journal.pone.0045162

**Published:** 2012-09-12

**Authors:** Andrea R. Josse, Bibiana Garcia-Bailo, Karina Fischer, Ahmed El-Sohemy

**Affiliations:** 1 Department of Nutritional Sciences, Faculty of Medicine, University of Toronto, Toronto, Ontario, Canada; 2 Institute of Food, Nutrition and Health, Department of Agriculture and Food Sciences, ETH Zurich, Zurich, Switzerland; Aligarh Muslim University, India

## Abstract

**Background:**

Hormonal contraceptive (HC) use may increase cardiometabolic risk; however, the effect of HC on emerging cardiometabolic and other disease risk factors is not clear.

**Objectives:**

To determine the association between HC use and plasma proteins involved in established and emerging disease risk pathways.

**Method:**

Concentrations of 54 high-abundance plasma proteins were measured simultaneously by LC-MRM/MS in 783 women from the Toronto Nutrigenomics and Health Study. C-reactive protein (CRP) was measured separately. ANCOVA was used to test differences in protein concentrations between users and non-users, and among HC users depending on total hormone dose. Linear regression was used to test the association between duration (years) of HC use and plasma protein concentrations. Principal components analysis (PCA) was used to identify plasma proteomic profiles in users and non-users.

**Results:**

After Bonferroni correction, 19 proteins involved in inflammation, innate immunity, coagulation and blood pressure regulation were significantly different between users and non-users (*P*<0.0009). These differences were replicated across three distinct ethnocultural groups. Traditional markers of glucose and lipid metabolism were also significantly higher among HC users. Neither hormone dose nor duration of use affected protein concentrations. PCA identified 4 distinct proteomic profiles in users and 3 in non-users.

**Conclusion:**

HC use was associated with different concentrations of plasma proteins along various disease-related pathways, and these differences were present across different ethnicities. Aside from the known effect of HC on traditional biomarkers of cardiometabolic risk, HC use also affects numerous proteins that may be biomarkers of dysregulation in inflammation, coagulation and blood pressure.

## Introduction

Hormonal contraceptives (HC) have been widely used globally since the middle of the 19^th^ century for reasons including the prevention of unintended pregnancy, the decreased risk of female (i.e. ovarian and endometrial) cancers, regulation of the menstrual cycle, control of acne and relief of pre-menstrual and menstrual symptoms [1]–[3]. Endogenous estrogens may protect against vascular disease and atherosclerosis in young women [4], [5], yet HC have also been linked to a greater risk of weight gain, cardiovascular disease, dyslipidemia, myocardial infarction, venous thromboembolism, and stroke [6]–[9]. Because of this, HC formulations have changed over the years and newer combinations of estrogen and progestin may confer less disease risk [10]. Since these medications remain widely used, and their physiological effects are widespread, it is important to further investigate how HC alter emerging disease risk pathways using both well known and novel clinical biomarkers.

Most studies of HC use have been conducted in young women and have assessed the effects of HC use on cardiovascular or cardiometabolic endpoints [9]–[15]. All of these studies found some evidence of increased risk among HC users [9]–[15]. Ethnocultural differences may also influence the effect of HC use on physiological pathways that are dysregulated during disease progression, and this may partly explain observed differences in rates of chronic disease across ethnocultural groups [16], [17]. Most [9], [10], [13]–[15], but not all [11], [12] of the studies mentioned were carried out in whites or mixed ethnic groups, and none compared the effects between ethnicities.

Novel technologies in the field of proteomics allow for the measurement of multiple high-abundance plasma proteins involved in several disease processes simultaneously [18], [19]. Such an approach yields a more comprehensive assessment of the plasma proteome, which may help to identify individuals who are at increased risk of disease, and define clinically relevant disease phenotypes [20], [21]. Proteomics may also be a useful tool to explore and identify new associations between plasma proteins and disease risk. There are more than 4000 proteins in human plasma, and they vary widely in concentration (e.g. albumin at ∼800 μmol/L, to Von Willebrand factor at ∼0.05 μmol/L) [22], [23]. Some of these proteins are established biomarkers of disease risk and have important roles in clinical diagnosis [24]. However, the individual functions of many other potentially important plasma proteins, including those involved in acute phase anti/pro inflammatory or anti/pro coagulatory processes, are less well defined, and their potential roles as putative biomarkers of disease risk are less well understood [25], [26].

Recent data from the Women's Health Initiative (WHI) suggested widespread effects of hormone replacement therapy (HRT) on the serum proteome of postmenopausal women [27], [28]. Use of these medications was shown to affect numerous proteins involved in physiologically important pathways, such as coagulation, inflammation, immunity and metabolism. These findings support a potential impact of HRT on disease risk through different mechanisms. However, the effect of HC, which differ in formulation from HRT, on the proteome in young women remains unknown. Given that HC use has been shown to increase the risk of cardiometabolic [9]–[15] and vascular disease (thromboembolism and stroke) [6], [7] and decrease the risk of certain cancers [2], [3], it is important to explore potential links between new and emerging disease risk biomarkers and HC use.

The objectives of this study were: 1) to determine whether HC use affects the concentrations of 55 high-abundance plasma proteins in an ethnoculturally diverse population of healthy young women, and 2) to investigate the effect of hormone dose and duration of hormone exposure on the same high-abundance proteins of the plasma proteome.

## Materials and Methods

### Ethics Statement

Participants gave written informed consent, and the protocol was approved by the Ethics Review Board of the University of Toronto, and conformed to standards for the use of human subjects in research as outlined in the Declaration of Helsinki. http://www.wma.net/en/30publications/10policies/b3/index.html

### Study population

Subjects were participants of the Toronto Nutrigenomics and Health study, a cross-sectional examination of men (*n* = 518) and women (*n* = 1,112) aged 20–29 years. Recruitment occurred between the fall of 2004 and the fall of 2010. Participants completed a general health and lifestyle questionnaire (GHLQ), a physical activity questionnaire, and a food frequency questionnaire, and gave a fasting blood sample. We excluded pregnant or breastfeeding women and individuals who were unable to provide a blood sample.

Of the initial 1,112 women recruited, 786 non-smokers had available proteomics data at the time this study was conducted. Of those 786, we excluded one individual with diabetes and two individuals with missing data on the variables included in the analyses. After exclusions, 783 individuals remained. Based on self-reported ancestry, women participating in the study were grouped into one of four ethnocultural groups: whites (*n* = 375), defined as those of European, Middle Eastern or Hispanic descent; East Asians (*n* = 282), defined as individuals of Chinese, Japanese, Korean, Filipino, Vietnamese, Thai or Cambodian descent; South Asians (*n* = 70), defined as those whose ancestors originated from the Indian subcontinent (India, Pakistan, Sri Lanka and Bangladesh); and others (*n* = 56), who included Aboriginal Canadian, Afro-Caribbean, or mixed-descent individuals. In this study, to ensure adequate sample sizes, the South Asian and Other categories were grouped together (*n* = 126).

### Hormonal contraceptive (HC) use

The use of HC was self-reported in the GHLQ, which included questions on type (e.g. the brand, and/or whether they were oral, trans-dermal or injected) and duration in years of HC use. Based on their responses, subjects were categorized as HC users (*n* = 240) or non-users (*n* = 543). Based on the type of medication used, users were further categorized into those taking HC with <1 mg or ≥1 mg of total hormone (estrogen + progesterone derived ingredients) per day, to ascertain whether different doses affected plasma proteomic biomarkers. Lastly, we assessed whether the duration of HC use in years affected the concentration of plasma proteins in the users.

### Anthropometric and physical activity measures

Anthropometric measurements, including height, weight, body mass index (BMI), systolic and diastolic blood pressure, were taken with the participant dressed in light clothing and no shoes, as described previously [19]. Physical activity, which was measured by questionnaire, was expressed as metabolic equivalent task (MET)-hours per week [19].

### Biochemical measurements

Blood samples were obtained from participants after a minimum 12-hour overnight fast. Subjects with temporary inflammatory conditions were asked to wait two weeks to provide blood. Samples were collected at LifeLabs medical laboratory services (Toronto, ON, Canada), and measurements of biomarkers of glycemic control, lipid metabolism, and the systemic inflammatory marker C-reactive protein (CRP) were performed on-site using standard procedures as described previously [29]. We calculated insulin resistance and β cell function using the homeostasis model assessment (HOMA) method [30].

### Plasma proteomic measurements

The concentrations of 63 high-abundance plasma proteins were measured at the University of Victoria – Genome British Columbia Proteomics Centre (Victoria, BC, Canada), using a multiple reaction monitoring (LC-MRM/MS) assay as described elsewhere [18], [19]. Of the 63 proteins measured, the intra-assay coefficients of variation (CV) for 9 of them were ≥15% [19]. Therefore, only 54 proteins with CVs <15% were included in this study.

### Statistical analysis

All statistical analyses were carried out using SAS (version 9.2; SAS Institute Inc, Cary, NC, USA). The α error was set at 0.05 and all reported *p*-values are two-sided. The Bonferroni correction for multiple testing was applied as necessary (55 tests, α = 0.05: *p*<0.0009). Subject characteristics were compared between HC users and non-users across all ethnicities using *χ^2^* tests for categorical variables and *t*-tests for continuous variables. We explored the individual associations between HC use and each of the 54 plasma proteomic biomarkers and CRP using general linear models (GLMs; ANCOVA) stratified by ethnicity and adjusted for age, waist circumference and physical activity. We assessed the distribution of continuous variables prior to analysis and log_e_- or square root-transformed those that were not normally distributed. In such cases, the *p*-values from models using transformed values are reported, but untransformed means and measures of spread are reported to facilitate interpretation. Biomarkers of glycemic control and lipid metabolism were compared between users and non-users using ANCOVA adjusted for age, waist circumference, physical activity and ethnocultural group. ANCOVA was also used (adjusted for age, waist circumference and physical activity) to determine the effect of total hormone dose (<1 mg versus ≥1 mg) on the 54 plasma proteins and CRP. We then used linear regression adjusted for the same covariates to assess, in users only and across 3 levels of use (non-users, <1 mg, and ≥1 mg), whether the duration of use of HC affected the plasma protein levels and whether a dose-dependent relationship was present, respectively.

Lastly, we used principal components analysis (PCA) to explore the relationship between HC use and the 54 plasma proteomic biomarkers and CRP. Using PCA, we identified plasma proteomic groups based on the concentrations of the measured proteins in HC users, as well as non-users. We obtained the principal components representative of the proteomic groups for both HC users and non-users through an orthogonal Varimax rotation that yielded independent principal components [31]. We used the Scree test and the Kaiser criterion of eigenvalues >1 to determine the individual principal components. For each principal component, inclusion of a particular protein in that component was determined based on a loading score criterion of ≥0.5. If one protein had a loading score of ≥0.5 for two principal components, the protein was included in both profiles.

## Results

### Study Population

Our study population was divided into 3 groups based on subjects' self-reported ethnocultural ancestry. In the white group, 166 subjects were HC users and 209 were non-users; in the East Asian group, 29 subjects were HC users and 243 were non-users; and in the South Asian/Other group, 35 subjects were users and 91 were non-users. **Table** **1** shows subject characteristics stratified by HC use across all ethnicities. Age and physical activity levels were significantly higher in users (**Table** **1**). HC use differed across ethnic groups, with nearly 45% of white women using HC, in contrast to 14% of East Asians and 28% of South Asians/Other. Plasma glucose, HOMA-β, triglycerides, free fatty acids, total cholesterol and high density lipoprotein (HDL) cholesterol were significantly higher in users versus non-users (**Table** **2**).

**Table 1:** Study participant characteristics stratified by hormonal contraceptive use.

	Non-users (*n* = 543)	Users (*n = *240)	*p-*value
Age (years)	22.4±2.5	23.0±2.4	0.0030
Ethnicity			
White	209 (55.7)	166 (44.3)	<0.0001
East Asian	243 (86.2)	39 (13.8)	
South Asian/Other	91 (72.2)	35 (27.8)	
Body mass index (kg/m^2^)*	22.3±3.4	22.6±3.3	0.1665
Waist circumference (cm)*	70.9±7.3	71.7±7.4	0.1240
Physical activity (met-h/wk)	7.4±3.1	8.0±2.7	0.0116

P-values are from *t*-tests for continuous variables and *χ^2^*-square tests for categorical variables.

Shown are untransformed means and standard deviations for continuous variables, and n(%) for categorical variables.

* Indicates variables that were log-transformed prior to statistical test.

**Table 2:** Biomarkers of glycemic control and lipid metabolism stratified by hormonal contraceptive use.

	Non-users (*n* = 543)	Users (*n* = 240)	*p*-value
Glucose (mmol/L)	4.7±0.4	4.7±0.3	0.0173
Fasting insulin (pmol/L)*	48.6±32.4	49.6±26.3	0.1436
HOMA-IR*	1.5±1	1.4±0.8	0.2677
HOMA-β*	113.7±80.0	123.7±70.3	0.0105
Triglycerides (mmol/L)*	0.9±0.5	1.2±0.4	<0.0001
Free fatty acids (umol/L)*	489.0±243.3	522.6±237.3	0.0101
Total cholesterol (mmol/L)	4.2±0.7	4.6±0.8	<0.0001
HDL cholesterol (mmol/L)*	1.6±0.4	1.8±0.4	<0.0001
LDL cholesterol (mmol/L)	2.2±0.6	2.3±0.7	0.0914
Total: HDL cholesterol ratio*	2.7±0.6	2.7±0.6	0.9059

P-values were adjusted for age, waist circumference, physical activity and ethnocultural group.

Shown are untransformed means and standard deviations.

* Indicates variables that were log-transformed prior to statistical test.

### Proteomics and CRP Analyses

Out of the 55 plasma proteins analyzed, 19 had significantly different concentrations at the Bonferroni level (*p*<0.0009) between users and non-users **(** **Table 3** **)**. These differences were consistent across ethnic groups. Of the 19 proteins, 16 had consistently higher concentrations in users (α_1_-Antitrypsin, Angiotensinogen, α_2_-HS-Glycoprotein, Apolipoprotein A-I, Apolipoprotein A-II Precursor, Apolipoprotein L1, CRP, Ceruloplasmin, Vitamin D Binding Protein, Coagulation Factor XIIa HC, Heparin Cofactor II, Kininogen-1, Plasminogen, Retinol-Binding Protein, Serum Amyloid P-Component, and Vitronectin) and three had consistently lower concentrations in users (Apolipoprotein E, Complement C1 Inactivator, Histidine-rich Glycoprotein). In addition to these 19 proteins, among whites, 16 other proteins had significantly different concentrations between users and non-users after Bonferroni correction, for a total of 35 out of 55. The majority of these proteins were elevated in users. We observed similar but less robust trends among the East Asians (21 out of 55 proteins were significantly different) and South Asians/Other (26 out of 55 proteins were significantly different) groups.

**Table 3:** Plasma proteomic and C-reactive protein (CRP) analyses stratified by users and non-users of hormonal contraception in three ethnocultural groups (Whites, East Asians and South Asians/Other).

	WHITES			EAST ASIANS			SOUTH ASIANS + OTHER			
	Non-Users (n = 209)	Users (n = 166)		Non-Users (n = 243)	Users (n = 39)		Non-Users (n = 91)	Users(n = 35)		
Plasma Protein (μmol/L)	Mean±SE‡	Mean±SE‡	*p*-value† *	Mean±SE‡	Mean±SE‡	*p*-value† *	Mean±SE‡	Mean±SE‡	*p*-value† *	DIRECTION relative to users
α_1_-Antitrypsin	10.58±0.17	14.52±0.27	***<.0001***	10.04±0.13	12.78±0.42	***<.0001***	11.15±0.25	14.08±0.48	***<.0001***	higher in users
Angiotensinogen	0.71±0.02	2.13±0.07	***<.0001***	0.67±0.01	1.76±0.13	***<.0001***	0.69±0.03	2.15±0.15	***<.0001***	higher in users
α_2_-HS-Glycoprotein	8.39±0.15	10.32±0.18	***<.0001***	8.31±0.1	9.56±0.32	***0.0003***	8.62±0.20	10.63±0.37	***<.0001***	higher in users
Apolipoprotein A-I	43.41±0.63	50.57±0.81	***<.0001***	43.99±0.62	50.66±1.61	***0.0004***	41.2±0.95	51.89±1.96	***<.0001***	higher in users
Apolipoprotein A-II Precursor	23.66±0.36	30.19±0.45	***<.0001***	23.61±0.31	29.35±0.98	***<.0001***	23.10±0.51	31.31±1.25	***<.0001***	higher in users
Apolipoprotein L1	0.36±0.01	0.6±0.02	***<.0001***	0.34±0.01	0.53±0.03	***<.0001***	0.41±0.02	0.58±0.03	***<.0001***	higher in users
C-Reactive Protein	0.92±0.17	2.69±0.26	***<.0001***	0.49±0.09	2.40±0.58	***<.0001***	1.36±0.29	2.25±0.38	***<.0001***	higher in users
Ceruloplasmin	2.12±0.05	3.69±0.09	***<.0001***	1.89±0.03	3.16±0.11	***<.0001***	2.26±0.07	3.82±0.21	***<.0001***	higher in users
D Vitamin Binding Protein	2.69±0.04	3.7±0.07	***<.0001***	2.56±0.03	3.38±0.11	***<.0001***	2.68±0.05	3.63±0.13	***<.0001***	higher in users
Coagulation Factor XIIa HC	0.28±0.01	0.40±0.01	***<.0001***	0.19±0.01	0.27±0.01	***<.0001***	0.22±0.01	0.39±0.02	***<.0001***	higher in users
Heparin Cofactor II	0.67±0.01	0.88±0.02	***<.0001***	0.63±0.01	0.76±0.03	***<.0001***	0.72±0.02	0.86±0.04	***0.0006***	higher in users
Kininogen-1	2.05±0.03	2.79±0.05	***<.0001***	1.94±0.02	2.46±0.07	***<.0001***	2.00±0.05	2.76±0.13	***<.0001***	higher in users
Plasminogen	1.15±0.02	1.50±0.02	***<.0001***	1.17±0.01	1.47±0.04	***<.0001***	1.21±0.02	1.54±0.06	***<.0001***	higher in users
Retinol-Binding Protein	0.86±0.02	1.17±0.02	***<.0001***	0.79±0.01	1.08±0.04	***<.0001***	0.77±0.02	1.13±0.05	***<.0001***	higher in users
Serum Amyloid P-Component	0.38±0.01	0.51±0.01	***<.0001***	0.35±0.01	0.56±0.03	***<.0001***	0.43±0.02	0.53±0.03	***0.0002***	higher in users
Vitronectin	3.53±0.05	4.65±0.08	***<.0001***	3.46±0.04	4.32±0.11	***<.0001***	3.70±0.08	4.69±0.17	***<.0001***	higher in users
Apolipoprotein E	0.5±0.01	0.43±0.01	***<.0001***	0.58±0.01	0.47±0.02	***0.0005***	0.57±0.02	0.41±0.02	***<.0001***	lower in users
Complement C1 Inactivator	4.73±0.08	3.74±0.1	***<.0001***	4.89±0.07	4.24±0.17	***0.0006***	5.04±0.12	3.92±0.24	***<.0001***	lower in users
Histidine-rich Glycoprotein	1.37±0.03	1.03±0.03	***<.0001***	1.43±0.03	1.14±0.07	***<.0001***	1.39±0.04	0.97±0.05	***<.0001***	lower in users
Afamin	0.24±0	0.29±0.01	***<.0001***	0.25±0.01	0.27±0.01	0.1485	0.25±0.01	0.30±0.01	***<.0001***	
α_1_-Acid Glycoprotein 1	1.97±0.05	1.59±0.04	***<.0001***	1.55±0.03	1.42±0.09	0.1009	1.97±0.07	1.48±0.08	***<.0001***	
Albumin	965.89±11.35	890.33±10.53	***<.0001***	976.09±9.52	896.57±12.8	*0.0019*	933.32±15.04	863.39±19.03	*0.0157*	
Apolipoprotein B-100	0.77 ± 0.02	0.93±0.02	***<.0001***	0.75±0.01	0.80±0.03	0.1799	0.77±0.03	0.95±0.04	***0.0006***	
Apolipoprotein C-III	2.28±0.05	3.05±0.07	***<.0001***	2.32±0.05	2.80±0.13	*0.0011*	2.16±0.09	2.89±0.15	***<.0001***	
Complement C3	18.98±0.32	21.86±0.35	***<.0001***	17.75±0.24	20.13±0.56	*0.0011*	21.10±0.59	22.44±0.85	0.1815	
Complement Factor B	1.41±0.03	1.57±0.03	***<.0001***	1.3±0.02	1.56±0.07	***0.0002***	1.66±0.05	1.66±0.06	0.8341	
Complement Factor H	0.60±0.01	0.65±0.01	***<.0001***	0.53±0.01	0.57±0.02	*0.0418*	0.66±0.02	0.66±0.02	0.6789	
Clusterin	1.5±0.02	1.64±0.03	***<.0001***	1.5±0.02	1.63±0.04	*0.0117*	1.52±0.03	1.68±0.06	*0.0188*	
Hemopexin	10.44±0.15	11.44±0.16	***<.0001***	9.53±0.13	10.71±0.24	*0.0014*	10.61±0.24	11.25±0.41	0.1937	
Inter- α-Trypsin Inhibitor HC	0.60±0.01	0.67±0.01	***<.0001***	0.62±0.01	0.68±0.02	*0.0059*	0.62±0.01	0.69±0.02	*0.0028*	
Prothrombin	0.56±0.01	0.62±0.01	***<.0001***	0.57±0.01	0.61±0.01	0.0641	0.58±0.01	0.60±0.02	0.6427	
Transferrin	12.41±0.21	14.57±0.25	***<.0001***	11.99±0.18	13.18±0.39	*0.008*	13.01±0.35	15.39±0.55	***0.0003***	
Transthyretin	5.38±0.09	5.97±0.09	***<.0001***	5.41±0.08	5.98±0.17	*0.0064*	4.97±0.11	5.84±0.21	***0.0003***	
Apolipoprotein A-IV	1.49±0.03	1.34±0.03	***0.0001***	1.32±0.02	1.29±0.05	0.7936	1.41±0.05	1.26±0.07	0.0649	
Antithrombin-III	3.59±0.05	3.38±0.04	***0.0008***	3.61±0.04	3.31±0.07	*0.0098*	3.54±0.06	3.30±0.09	*0.0292*	
α_1B_-Glycoprotein	1.72±0.03	1.89±0.05	*0.002*	1.62±0.03	1.78±0.09	0.0864	1.64±0.06	1.84 ± 0.12	0.122	
Apolipoprotein C-I	3.31±0.07	3.51±0.06	*0.0074*	3.18±0.05	3.43±0.14	0.1587	3.13±0.10	3.36±0.13	0.1626	
L-Selectin	0.08±0	0.07±0	*0.0089*	0.07±0.01	0.06±0.01	*0.0059*	0.07±0.01	0.07±0.01	0.0537	
Fibrinogen γ Chain	9.54±0.3	10.27±0.34	*0.0139*	9.46±0.24	11.09±1.23	0.1462	10.13±0.38	10.61±0.5	0.3454	
Fibrinogen β Chain	9.59±0.26	10.27±0.33	*0.0192*	9.49±0.23	10.71±0.97	0.1513	10.06±0.33	11.1±0.58	0.1364	
Fibrinogen α Chain	11.99±0.38	12.94±0.49	*0.0205*	12.01±0.33	14.25±1.88	0.1225	12.36±0.44	13.9±0.72	0.0979	
Fibrinopeptide A	7.17±0.19	7.56±0.21	*0.0305*	6.99±0.16	8.07±0.85	0.1418	7.60±0.24	8.09±0.38	0.3526	
Gelsolin, isoform 1	1.19±0.02	1.11±0.02	*0.0343*	1.17±0.02	1.05±0.04	***0.0005***	1.21±0.03	1.18±0.05	0.6169	
α_2_-Antiplasmin	1.92±0.03	1.99±0.03	*0.0344*	1.91±0.03	1.95±0.04	0.369	1.93±0.04	2.03±0.07	0.2039	
Zinc- α_2_-Glycoprotein	1.02±0.03	1.08±0.03	0.0561	0.96±0.02	1.04±0.05	0.233	1.01±0.04	1.09±0.06	0.2064	
α_2_-Macroglobulin	6.1±0.12	5.92±0.15	0.0972	6.19±0.1	5.79±0.19	0.2542	6.25±0.18	6.03±0.22	0.7205	
α_1_-Antichymotrypsin	3.5±0.06	3.35±0.06	0.1129	3.22±0.05	3.19±0.1	0.8063	3.63±0.09	3.17±0.13	*0.0016*	
Apolipoprotein D	0.35±0.01	0.34±0.01	0.1283	0.33±0.01	0.32±0.01	0.4027	0.36±0.01	0.33±0.01	0.0988	
Complement C9	2.8±0.05	2.69±0.06	0.1585	2.71±0.05	2.84±0.16	0.6286	3.17±0.10	2.60±0.10	*0.0017*	
Complement C4 β Chain	1.36±0.03	1.4±0.04	0.1646	1.36±0.03	1.38±0.08	0.9182	1.66±0.07	1.65±0.12	0.9227	
Complement C4 γ Chain	1.49±0.03	1.53±0.04	0.2421	1.51±0.04	1.51±0.08	0.7504	1.83±0.08	1.85±0.13	0.904	
Adiponectin	0.08±0	0.07±0	0.2474	0.06±0.01	0.06±0.01	0.6549	0.06±0.01	0.06±0.01	0.5314	
Fibronectin	0.65±0.08	0.62±0.1	0.4056	0.59±0.07	0.73±0.22	0.8263	0.50±0.04	0.65±0.10	0.5252	
Haptoglobin β Chain	11±0.35	10.45±0.36	0.6736	9.86±0.32	9.21±0.91	0.3018	13.38±0.70	9.48±1.09	***<.0001***	
β_2_-Glycoprotein I	2.83±0.05	2.80±0.05	0.8658	2.62±0.03	2.61±0.06	0.9237	2.77±0.06	2.73±0.14	0.5768	

Bold and Italic  =  Significant at the Bonferroni level; Italic  =  significant but not at the Bonferroni level.

* Adjusted for age, waist circumference and physical activity.

† Untransformed, log transformed or square route transformed.

‡ Unadjusted and untransformed means and standard errors.

The concentrations of two proteins were significantly different between users and non-users in only one ethnocultural group (**Table 3**). These were Gelsolin isoform 1 in East Asians and Haptoglobin β Chain in South Asians/Other. Results for plasma protein concentration differences among ethnocultural groups (men and women) not stratified by HC use have been published elsewhere [19].

### Duration of Hormonal Contraceptive (HC) Use

We assessed whether the duration of HC use, measured in years, affected plasma protein concentrations in users only across all ethnocultural groups. Six proteins changed significantly (*p*<0.05) with increasing duration of HC use. Three proteins were significantly lower with increased duration of use: Complement C9 ([β estimate ± SE, r^2^, *p*-value] −0.178 ±0.07, 0.06, 0.04), α_2_-Macroglobulin (−0.32±0.15, 0.07, 0.03), and α_1_-Antichymotrypsin (−0.15±0.07, 0.11, 0.03). Three others were significantly higher with increased duration of use: Apolipoprotein A-II Precursor (1.31±0.54, 0.07, 0.02), Apolipoprotein C-III (0.14±0.08, 0.04, 0.05), and Transferrin (0.54±0.29, 0.06, 0.05). None of these differences in protein concentration remained significant after Bonferroni correction.

### Dose of Hormones in Contraceptives (HC)

We assessed whether the dose of total HC hormone affected plasma protein levels. Subjects reported using twelve different types of HC. We divided them by total hormone concentration into three groups: 0 mg/d (n = 531 non-users), <1 mg/d (*n* = 141 users) and ≥1 **c**mg/d (*n* = 52 users). We excluded subjects (*n* = 59) from this analysis if they were getting hormone injections every few months since it was difficult to ascertain a daily hormonal exposure, if they were HC users but classified their type of medication as “other”, if they classified themselves as non-users but reported a medication type (i.e. misreporting), or if they provided no information at all on HC use. Seven proteins were significantly different between the two groups of HC users. Four proteins were significantly higher in the <1 mg compared to the ≥ 1 mg group, respectively: Apolipoprotein L1 ([mean ± SE] 0.62±0.02 vs. 0.54±0.03, *p*<0.003), Albumin (901.40±10.21 vs. 845.57±16.85, *p*<0.009), Serum Amyloid P-Component (0.55±0.01 vs. 0.50±0.02, *p*<0.02), α_1_-Antitrypsin (14.73±0.28 vs. 13.83±0.46, *p*<0.05). Three were lower in the <1 mg compared to the ≥1 mg group, respectively: Complement C4 γ Chain (1.52±0.05 vs. 1.75±0.08, *p*<0.03), Complement C4 β Chain (1.40±0.04 vs. 1.58±0.07, p<0.04), Histidine-rich Glycoprotein (0.98±0.03 vs. 1.08±0.05, *p*<0.05). None of the proteins remained significant after Bonferroni correction. We then examined whether these associations were dose-dependent by comparing the two levels of hormone dose (<1 mg/d and ≥1 mg/d) to no hormone dose (i.e. non-users). No dose-response effects were observed.

### Principal Components Analysis

**Figure 1:**
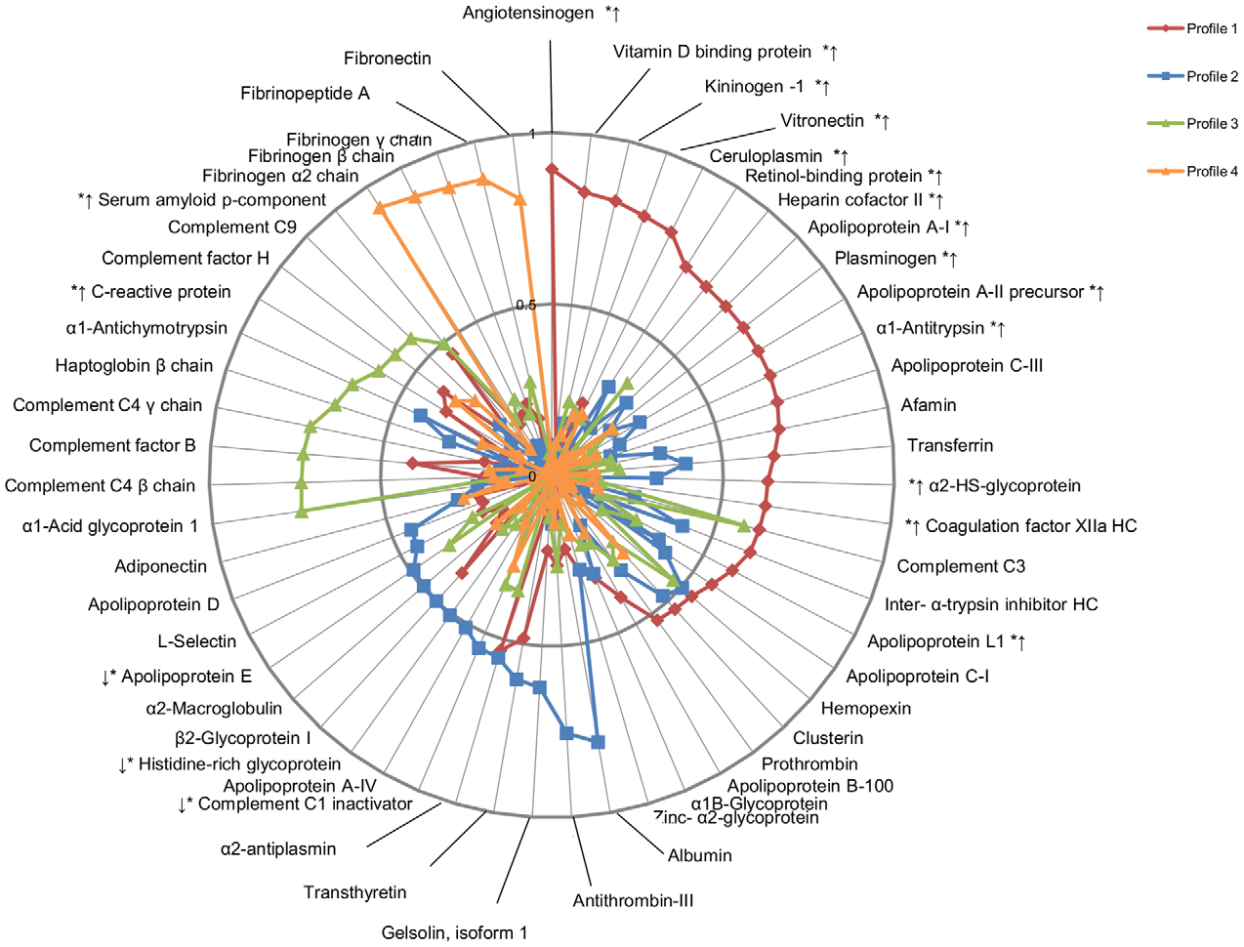
Principal components analysis (PCA) in users of HC. Four independent proteomic profiles were identified, based on a loading score criterion of >0.5. Profile 1 consisted of primarily positive acute phase reactants, while profile 2 comprised mainly negative acute phase reactants. Profile 3 consisted of complement system components and acute phase proteins, and Profile 4 represented primarily proteins involved in coagulation. * designates the 19 proteins that were significantly different between users and non-users in the whole study population. The arrows designate the direction of the difference (i.e. higher or lower levels of the protein) with respect to non-users.

Four distinct proteomic profiles were identified among HC users (**Figure** **1**). Profile 1 included 24 proteins, most of which were positive acute phase reactants involved in inflammatory and blood pressure-related processes, such as Complement C-3, α_1_-Antitrypsin and Angiotensinogen. Fourteen of these proteins were significantly higher in users than in non-users (**Figure** **1**). Profile 2 was comprised of 10 anti-inflammatory and anti-coagulatory negative acute phase proteins. Two of these proteins were significantly lower in users than non-users (Complement C1 Inactivator, Histidine-rich Glycoprotein). Profile 3 consisted of 11 innate immunity-related complement proteins, adaptive immunity-related acute phase proteins and CRP. Two of these proteins (CRP and Serum Amyloid P-Component) were significantly higher in users than non-users. Profile 4 consisted of 5 proteins that were exclusively related to coagulation, and none were significantly different between users and non-users. The variance explained by profiles 1-4 in HC users was 13.4%, 6.9%, 6.6% and 5.0%, respectively, and together, these principal components explained 32% of the observed variance in the data set. Three proteins (Complement C3, Hemopexin and α_2_-Antiplasmin) had loading scores of >0.5 for two principal components (**Figure** **1**) and were, therefore, included in both components. Eight proteins (Apolipoprotein B-100, α_1B_-Glycoprotein, Zinc-α_2_-Glycoprotein, L-Selectin, Adiponectin, Apolipoprotein D, α_2_-Macroglobulin, Apolipoprotein E) had loading scores <0.5 for each principal component and, therefore, were not included in any profile.

**Figure 2:**
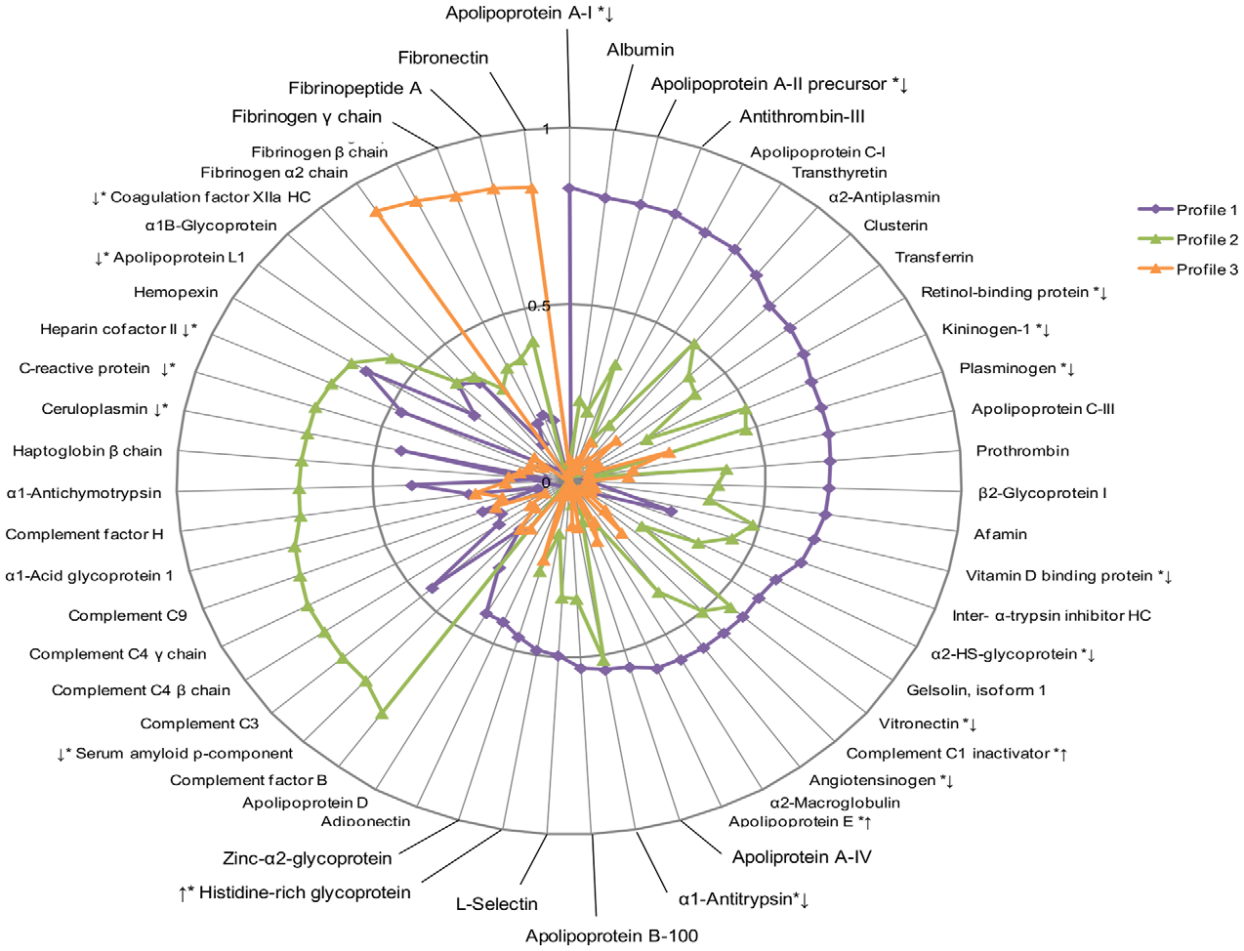
Principal components analysis (PCA) in non-users of HC. In contrast to HC users, only three independent proteomic profiles were identified among non-users of HC, based on a loading score criterion of >0.5. No profile was characteristically representative of a negative or positive acute phase response. * designates the 19 proteins that were significantly different between users and nonhyphen;users in the whole study population. The arrows designate the direction of the difference (i.e. higher or lower levels of the protein) with respect to users.

Three distinct proteomic profiles were identified among non-users of HC (**Figure 2**). Profile 1 included 29 proteins from several physiologic pathways including positive acute phase proteins involved in inflammation and blood pressure (e.g. Plasminogen, Complement C4 and Angiotensinogen), as well as negative acute phase proteins involved in downregulating inflammation and coagulation (e.g. Transthyretin and Kininogen-1). Ten of these proteins were significantly lower in non-users than in users, and two were significantly higher in non-users than in users. Profile 2 was comprised of 19 innate immunity-related complement proteins, innate and adaptive immunity-related acute phase proteins, and CRP. Five of these proteins were significantly lower in non-users than in users. Profile 3, like profile 4 in the users, contained 5 proteins exclusively related to coagulation, and none were significantly different between users and non-users. The variance explained by profiles 1–3 in HC non-users was 15.8%, 12.1% and 4.5%, respectively, and together, these principal components explained 32% of the observed variance in the data set. Five proteins (Hemopexin, α_2_-Antiplasmin, Vitronectin, Complement C1 Inactivator and α_1_-Antitrypsin) had loading scores of >0.5 for two principal components (**Figure 2**) and were, therefore, included in both components. Seven proteins (L-Selectin, Histidine-rich Glycoprotein, Zinc- α_2_-Glycoprotein, Adiponectin, Apolipoprotein D, α_1B_-Glycoprotein, and Coagulation Factor XIIa HC) had loading scores <0.5 for each principal component and were, therefore, were not included in any profile.

## Discussion

This study assessed the effects of HC use on both novel plasma proteomic biomarkers and traditional cardiometabolic risk factors in an ethnoculturally diverse population of young women. Our results suggest that HC use modulates the levels of multiple high abundance plasma proteins belonging to pathways that become dysregulated during disease progression. Indeed, 19 of the 55 plasma proteins measured were significantly different between users and non-users, and this effect was observed consistently across three distinct ethnocultural groups. The concentrations of most of these proteins were higher in users, including those that are established biomarkers of cardiovascular disease risk, such as CRP [24] and angiotensinogen [32]. Other proteins involved in (but not limited to) coagulation, such as Coagulation Factor XIIa HC, Heparin Cofactor II, Plasminogen and Vitronectin, were all higher in users, suggesting that HC use may also modulate disease risk *via* this pathway [33], [34]. While 19 proteins showed consistent differences across all ethnicities, an additional 9 proteins were significantly different only in whites. As well, Gelsolin was only different between users and non-users in East Asians, while Haptoglobin β Chain was only different between users and non-users in South Asians/Other. These results may indicate differential responses to HC use among ethnicities; however, our power to detect differences in some of the other ethnic groups may have been limited due to decreased sample sizes, and in particular, a lower proportion of HC users in East Asians and South Asians/Others.

Previous work examined the effects of HRT consisting of estrogens (conjugated equine estrogens; CEE) or CEE plus progestin (medroxyprogesterone acetate; CEE + P), on the serum proteome in postmenopausal women participating in the WHI [27], [28]. Together, these studies identified approximately 100 proteins whose levels were affected by the use of either type of hormone replacement medication. The proteins identified were involved in coagulation, inflammation, metabolism, the immune response, and other physiologically important pathways. In the present study, of the 19 proteins associated with HC use, 10 proteins (Angiotensinogen, α_2_-HS-Glycoprotein, Apolipoprotein A-II precursor, Ceruloplasmin, D Vitamin Binding Protein, Coagulation Factor XIIa HC, Kininogen-1, Plasminogen, Retinol-Binding Protein, and Vitronectin) showed similar associations with HRT in the WHI [27], [28]. Several differences exist between the present study and the WHI studies. In addition to fundamental physiological differences between younger and older (postmenopausal) women, as well as ethnic differences in the study samples, the formulation of hormone medications used in each study differed. HC generally consist of a combination of synthetic ethinyl estradiol and progestins [35], while the HRT used in the WHI were equine estrogens (estrone, equilin and equilenin), either alone or in combination with progestin [27], [28]. Furthermore, the WHI studies employed a different proteomic profiling method that allowed for the assessment of a greater number of proteins, but in doing so, prior to analysis, they depleted the samples of some highly abundant proteins, such as Albumin, Haptoglobin, and α_1_-Antitrypsin. Despite these differences, the considerable overlap in identified proteins and physiologic pathways affected by hormone medications between our study and the WHI studies provides compelling evidence that the use of estrogenic hormones, regardless of formulation or life stage, has marked effects on the plasma/serum proteome.

We also investigated whether the dose of daily hormone or the duration of HC use were associated with plasma protein concentrations. Two complement proteins (Complement C4 γ Chain and Complement C4 β Chain) had higher concentrations in those with greater hormone exposure, suggesting that HC dose may affect pathways involved in innate immunity. However, the differences were no longer significant after correcting for multiple comparisons. With regards to duration of HC use, our results suggest that years of exposure to HC may affect cholesterol metabolism, with increased levels of Apolipoprotein A-II Precursor and Apolipoprotein C-III, yet, again, differences were no longer significant after Bonferroni correction. In agreement with our results, a population-based retrospective analysis of a multicultural North American cohort also found no association between long-term oral contraceptive use and the prevalence of metabolic syndrome, measures of glycemic control or lipid metabolism [10]. Finally, our PCA analysis illustrated that plasma proteins clustered differently among users and non-users of HC. Overall, our results show that plasma protein concentrations indeed differed between users and non-users of HC in the direction of increased inflammation and dysregulation of certain pathways involved in coagulation and innate immunity in users. Our findings corroborate previous research on HC use and cardiometabolic disease risk [9]–[15], but also highlight potential novel effects of HC on other disease risk pathways. To our knowledge, the present study is the first to report the widespread effects of HC on the plasma proteome.

We observed a greater number of associations between HC and plasma proteomic biomarkers among whites than the other ethnic groups. We also noted ethnic-specific differences between users and non-users for certain proteins (**Table 3**). These observations may be partly a result of the difference in sample sizes between the ethnic groups. Nonetheless, this is the first study to assess the association between these proteins and HC use across ethnicities. Gelsolin is a member of the actin scavenging system with anti-apoptotic and anti-inflammatory properties [36]. Low levels of Gelsolin have been identified as a potential colorectal cancer biomarker in a Chinese population [37]. Haptoglobin is a positive acute phase reactant with antioxidant properties that regulates the pro-oxidant activity of hemoglobin [38]. The lower levels of Gelsolin and Haptoglobin among East Asian and South Asian/Other HC users, respectively, support the view that HC may contribute to the dysregulation of physiological processes in these groups. Whether the observed differences translate into ethnic-specific effects of HC on cardiometabolic disease remains to be elucidated. Indeed, a common polymorphism in the Haptoglobin gene has been associated with increased cardiometabolic and autoimmune disease risk [39], and Haptoglobin genotypes have been shown to modify the relationship between dietary intake of vitamin C, an antioxidant, and circulating levels of ascorbic acid [40].

PCA revealed different plasma proteomic profiles among HC users and non-users. In users, four profiles were identified (**Figure 1**). Profile 1 consisted of primarily positive acute phase reactants, such as Complement C3 and Ceruloplasmin, while profile 2 comprised mainly negative acute phase reactants, such as Transthyretin and Albumin [25], [41]. These distinct groupings suggest a different acute phase response in HC users, which may translate into increased disease risk. In contrast, only three proteomic profiles were identified in non-users (**Figure 2**), and no profile was characteristically representative of a negative or positive acute phase response. Overall, this indicates a different pattern of plasma protein clustering between users and non-users of HC. Future studies assessing the effects of HC on particular plasma proteins or pathways may shed light on the biological implications of this finding.

This study had some limitations. First, information on type of HC (i.e. different generation formulations) was not assessed. Earlier contraceptive formulations, which contained higher levels of estradiol and no progestins, were associated with a different disease risk profile than later HC formulations [10]. We did not distinguish between HC generation when examining the associations between HC use and individual proteins or proteomic profiles. However, given the younger age of the study participants, and noting the brands of contraceptives they were taking, the majority of users reported taking third generation medications. Another limitation is the cross-sectional nature of the study, which prevents establishing causality for any of the observed associations. In addition, the small sample size of the non-white ethnic groups, particularly the South Asian and Other category, may have led to a lack of sufficient statistical power to adequately assess the effect of HC in these individual groups. Finally, although we adjusted for a number of covariates, residual confounding may have affected some of the observed results.

In summary, we observed associations between HC use and the concentrations of 19 plasma proteomic biomarkers, as well as cardiometabolic biomarkers of disease risk, across distinct ethnocultural groups, in a population of healthy young women. HC use was associated with higher concentrations of numerous proteins along inflammatory, coagulatory and immune related pathways that become dysregulated during disease progression. By examining the plasma proteome, this study sheds some light on possible novel pathophysiologic links between HC use and disease beyond their established effect on the cardiovascular system. In conclusion, HC use has a profound and robust effect on the proteins investigated in this plasma proteomic panel, and hence, may be associated with increased disease risk through novel pathways and mechanisms.
